# Bilateral endoscopic technique for left atrial appendectomy and robot-assisted mitral valve repair

**DOI:** 10.1093/icvts/ivab250

**Published:** 2021-09-18

**Authors:** Ryuta Seguchi, Toshiya Ohtsuka, Norihiko Ishikawa, Go Watanabe

**Affiliations:** Department of Cardiovascular Surgery, NewHeart Watanabe Institute, Tokyo, Japan

**Keywords:** Left atrial appendage, Robotic mitral valve repair, Stapler appendectomy

## Abstract

We report a bilateral thoracoscopic technique in which robot-assisted mitral valve repair was achieved concomitantly with stapler division of the large left atrial appendage. The patient was a 65-year-old male with severe mitral regurgitation, paroxysmal atrial fibrillation and a large-sized atrial appendage. Closure of the appendage was completed off-pump using a left thoracoscopic stapler-division technique previous to right thoracoscopic robot-assisted mitral valve repair and cryoablation. Complete closure of the appendage was confirmed in thoracoscopic views. The bilateral thoracoscopic technique could be preferable for the minimally invasive treatment of mitral valvular disease and concomitant large-sized atrial appendage management.

## INTRODUCTION

Robot-assisted totally endoscopic mitral valve repair has been developed as an option for mitral valve procedures [1]. Atrial fibrillation is not uncommon in such cases and concomitant closure of the left atrial appendage (LAA) will significantly prevent stroke [2]. However, surgical risks of incomplete suture, Haemorrhage and circumflex artery damage underlie in the intracardiac direct suturing of oversized LAA. We herein report a bilateral thoracoscopic technique in which robot-assisted mitral valve repair was achieved with stapler division of the LAA.

## CASE REPORT

A 65-year-old male with severe mitral regurgitation caused by P3 prolapse, accompanied with atrial fibrillation, was admitted for minimally invasive surgery. Computed tomography revealed a bulky LAA, 49 mm in diameter, with its large ostium adjacent to the circumflex coronary artery. The respiratory function was normal.

The patient laid on the supine position and general anaesthesia was delivered through a double-lumen endo-tracheal tube.

**Figure 1:**
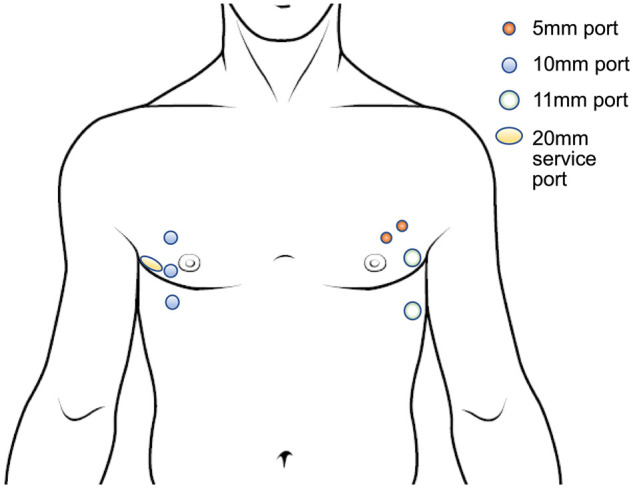
Scheme of 8 keyhole skin incisions.

First, left thoracoscopic off-pump stapler division of the LAA was performed. A four-port system was used (Fig. 1): a 5-mm port at the third intercostal space on the mid-clavicular line for the 5-mm, 30-degree-angled rigid scope; 11-mm port at the fifth intercostal space on the mid-axillary line for the stapler (ECHELON FLEX Powered ENDOPATH Stapler60, Ethicon, Raritan, New Jersey, USA); and the other 2 ports for assisting devices. A 6-cm pericardiotomy was made 10 mm posterior to the phrenic nerve. The stapler test-clamped the base of the LAA to check for possible haemodynamic collapse due to occlusion of the circumflex artery; electrocardiography and transoesophageal echocardiography showed no changes. The LAA was stapled with perfect haemostasis, leaving no stumps (Video 1).

**Figure 2:**
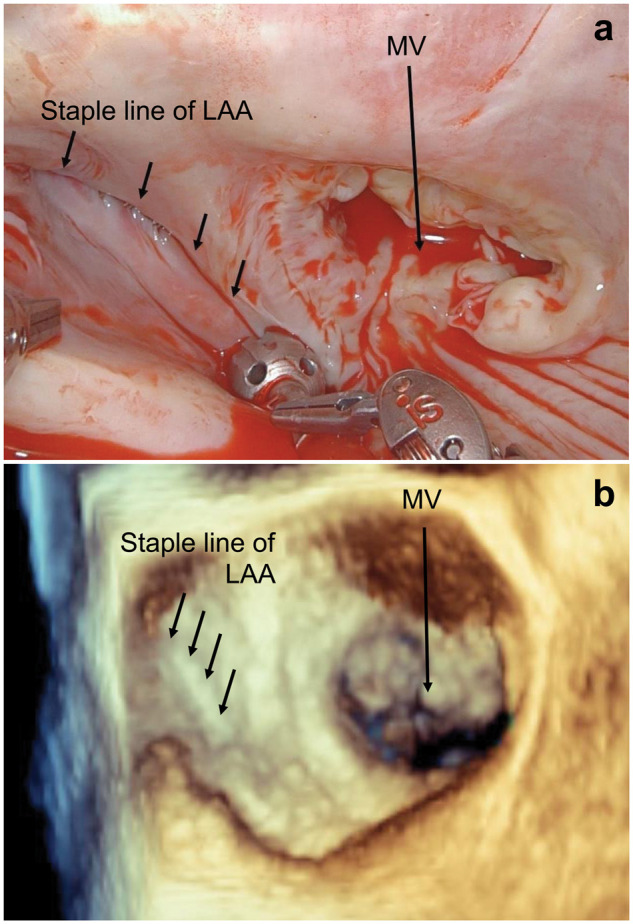
Image of complete left atrial appendage closure without remnant stump. (**a**) Thoracoscopic view. (**b**) Transoesophageal echocardiographic image after weaning from cardiopulmonary bypass. LAA: left atrial appendage; MV: mitral valve.

Second, cardiopulmonary bypass (CPB) was established via cannulation into the femoral artery and the jugular and femoral veins, and right thoracoscopic robot-assisted procedure was performed through 4 ports using the robot (daVinci Surgical System, Intuitive, Sunnyvale, California, USA). The flatness of the closure without stump was confirmed endocardially (Fig. 2). The mitral valve was repaired by neochordae implantation, ring annuloplasty and edge-to-edge techniques. The left atrial cryoablation was performed with the designated probe (CryoICE, AtriCure, Mason, Ohio, USA); box isolation of the pulmonary veins; and mitral valve-isthmus ablation (Video 2). The operation, CPB and aortic cross-clamp time were 236, 107 and 63 min, respectively.

Transoesophageal echocardiography revealed a flat closure of the LAA and no mitral regurgitation. No paroxysmal atrial fibrillation was observed postoperatively in the 10-month follow-up period.

## COMMENT

Patients having large LAA are at a higher risk of cardiogenic stroke [3]. However, endocardial suture-closure of the oversized LAA ostium poses incomplete closure problem with Haemorrhage and may cause a damage to the adjacent circumflex artery. An LAA clip or a stapler can be introduced through a right thoracoscopic port to the base of the LAA, passing through the transverse sinus under on-pump situation. However, this approach is not easy when the LAA tissue is large. By contrast, although additional ports are required, the stapler technique through left thorax is swiftly achievable regardless of LAA size [4].

Nevertheless, patient selection should be carefully considered. Since the lungs are collapsed alternately, patients with poor respiratory function may not tolerate. Although the CPB time could be saved, bilateral lung collapse would damage respiratory function. The left thoracoscopic part should be shortened as much as possible.

In the present case, left atrial cryoablation was performed, although full Cox-maze IV procedure is recommended to achieve sinus rhythm [5]. Since the atrial fibrillation was paroxysmal, left atrial cryoablation was chosen to shorten the CPB time and save the right atriotomy. If the fibrillation recurs, touch-up transcutaneous ablation could be a choice.

## CONCLUSION 

In conclusion, the stated technique is a preferable option for mitral valve insufficiency and atrial fibrillation possessing oversized LAA. The methodology would be adaptable to any mitral procedures through similar minimally invasive approach.

**Conflict of interest:** none declared.

### Reviewer information

Interactive CardioVascular and Thoracic Surgery thanks Ovidio A. García-Villarreal and the other, anonymous reviewer(s) for their contribution to the peer review process of this article.
